# “Zoom”ing to the Kitchen: A Novel Approach to Virtual Nutrition Education for Medical Trainees

**DOI:** 10.3390/nu15194166

**Published:** 2023-09-27

**Authors:** Justin A. Charles, Nathan I. Wood, Stephanie Neary, Jorge O. Moreno, Lindsey Scierka, Benjamin Brink, Xiwen Zhao, Katherine A. Gielissen

**Affiliations:** 1 Department of Family Medicine and Public Health, UC San Diego Health, San Diego, CA 92093, USA; 2 Department of Internal Medicine, Yale University School of Medicine, New Haven, CT 06520, USA; nathan.wood@yale.edu (N.I.W.); jorge.moreno@yale.edu (J.O.M.); lindsey.scierka@yale.edu (L.S.); 3 Physician Assistant Online Program, Yale University School of Medicine, New Haven, CT 06520, USA; stephanie.neary@yale.edu; 4 Department of Internal Medicine, Brown University-Rhode Island Hospital, Providence, RI 02912, USA; 5 Yale Center for Analytical Sciences, Yale University School of Public Health, New Haven, CT 06511, USA; xiwen.zhao@yale.edu; 6 Division of General Internal Medicine, Emory University School of Medicine, Atlanta, GA 30303, USA; kgielis@emory.edu

**Keywords:** teaching kitchen, culinary medicine, cooking, plant-based diet, food, nutrition, medical education, curriculum, virtual learning

## Abstract

While nutritional interventions are first-line therapy for many chronic diseases, most medical trainees receive minimal nutrition education, leaving them unprepared to address nutritional issues with patients. An interactive, single-session, virtual nutrition curriculum was taught online to 80 physician assistant (PA) students. Topics included plant-based nutrition, dietary history-taking and counseling, and culinary medicine. Students were surveyed before, immediately after, and four weeks after the curriculum to assess changes to nutrition-related knowledge, attitudes, confidence, and personal dietary behaviors. Seventy-three PA students (91%) completed the pre-survey, 76 (95%) completed the post-survey, and 42 (52.5%) completed the delayed post-survey. Knowledge scores increased immediately post-intervention (48.9% to 78.9%; *p* < 0.001) and persisted four weeks later (78.9% to 75.8%; *p* = 0.54). Post-intervention, students felt more confident in dietary history-taking (55% vs. 95%; *p* = 0.001) and nutrition counseling (53% vs. 84%; *p* = 0.003) and agreed that dietary changes alone could reverse type 2 diabetes (74% vs. 97%; *p* = 0.027) and coronary artery disease (66% vs. 92%; *p* = 0.039). Curricula using virtual teaching kitchens may be a scalable approach to nutrition education for medical trainees.

## 1. Introduction

An unhealthy diet is the leading global risk factor for non-communicable chronic disease, morbidity, and mortality [1,2]. Adopting a healthy lifestyle, including a diet low in meat and high in fruits, vegetables, and whole grains, can prevent up to 80% of chronic diseases and add up to 12–14 years of life expectancy [3,4]. While guidelines recommend nutrition interventions as first-line therapy for chronic disease, many clinicians view them as adjunctive treatment [5,6]. A whole-food, plant-based (WFPB) diet, which emphasizes minimally processed vegetables, fruits, whole grains, legumes, nuts, and seeds and minimizes or eliminates animal products, has the potential not only to prevent and treat but even reverse chronic diseases, including coronary artery disease (CAD), type 2 diabetes mellitus (T2DM), hypertension, hyperlipidemia, and obesity [7,8,9,10,11,12,13].

Despite the known health impacts of nutrition, nutrition education is lacking among health professions trainees, including physician assistants (PAs). The Accreditation Standards for Physician Assistant Education do not require specific nutrition training [14]. In a 2022 survey of 353 currently practicing PAs, most PAs lacked sufficient knowledge, confidence, and skills to deliver optimal nutritional care for chronic disease [15]. The majority were dissatisfied with the current PA nutrition curricula. While medical education leadership agrees that more formal nutrition curricula are necessary [16,17], there is no clear consensus on how best to implement nutrition education for healthcare professionals [14].

Culinary medicine, taught either in-person or in virtual teaching kitchens, is an evidence-based medical education and patient care field that combines nutrition science and culinary arts to promote wellness and prevent and treat chronic disease [18,19]. Teaching kitchens, both physical and virtual, serve as learning laboratories to promote health and wellness, culinary skill development, nutrition knowledge acquisition, and behavior change [20,21]. Culinary medicine shows promise as a successful modality for teaching nutrition due to its hands-on, interactive approach and may have superior effectiveness than more traditional nutrition education due to its alignment with experiential learning theory [22,23,24,25]. Three recently published scoping reviews on culinary medicine curricula for medical students [26,27] and, more broadly, for health profession trainees [28] have highlighted the beneficial effects of culinary medicine education on nutrition knowledge, skills, and counseling ability and personal dietary behaviors. By “training the trainers”, culinary medicine is uniquely poised to address the lack of clinicians proficient in clinical nutrition [29], improve learners’ personal dietary habits, and promote efficacy in counseling patients on nutrition and positive behavior change [30,31,32,33,34].

To address the lack of formal nutrition curricula in the Yale Physician Assistant Online Program (YPAOP), we designed an interactive virtual nutrition curriculum focused on evidence-based nutrition approaches using interactive learning methods. Primary outcomes included changes in nutrition knowledge before and after the educational intervention. Secondary outcomes included self-reported changes in attitudes and skills of PA students after their participation.

## 2. Materials and Methods

### 2.1. Curriculum Design

Kern’s six-step approach to curriculum development in medical education was used to design, implement, and evaluate this nutrition curriculum [35]. This approach is well described in the medical education literature and involves performing general and targeted needs assessments, creating relevant goals and objectives, choosing appropriate educational strategies to achieve those goals, implementing and refining the curriculum, conducting learner evaluation, and soliciting feedback. Our targeted needs assessment revealed that 30 min of formal curricular time was dedicated to nutrition education in the current YPAOP curriculum.

The goal of the curriculum was to deliver an interactive, evidence-based, and practical session that would engage trainees and provide them with the knowledge, skills, and attitudes necessary to address nutrition in patients with chronic disease meaningfully. These goals informed the development of cognitive, affective, and skills-based curricular learning objectives, listed in Supplementary S1. These learning objectives focused on (1) knowledge of evidence-based nutrition needed to advise patients on the prevention and management of chronic diet-related disease, (2) skills to assess a patient’s nutritional status via dietary history-taking and to counsel patients effectively, and (3) the basic culinary knowledge and skills needed to prepare an inexpensive, quick, health-promoting, plant-based meal.

To develop learning objectives, we reviewed published nutrition curricula and consulted with medical education, nutrition, and lifestyle medicine experts to identify relevant content and effective teaching modalities. The curriculum was divided into three one-hour modules: a didactic session (“Using a Plant-Based Diet for Chronic Disease Prevention and Treatment”), an interactive activity related to performing a dietary history and nutrition counseling (“The 5 *A*s of Behavior Change and Performing a 24-h Dietary Recall”), and an introductory culinary medicine session of hands-on cooking in a virtual teaching kitchen (“A Culinary Medicine Crash Course”). These modules are described in further detail below.

Funding for the curriculum was obtained through the Yale Office Based Medicine Scholarship and Innovation Fund and the American College of Lifestyle Medicine (ACLM) Trainee Research Scholarship and Grant.

#### 2.1.1. Using a Plant-Based Diet for Chronic Disease Prevention and Treatment

A one-hour didactic session was developed to introduce the principle of the dietary spectrum, highlighting the Standard American Diet, Dietary Approaches to Stop Hypertension (DASH) diet, Mediterranean diet, and WFPB diet [36]. The content and design of the presentation were informed by the literature. The presentation was iteratively edited with feedback from the study authors and outside content experts to ensure the content was relevant, accurate, and met the stated learning objectives.

#### 2.1.2. Nutrition Counseling in Primary Care: The 5 As of Behavior Change and Performing a 24-h Dietary Recall

A one-hour module was developed to teach learners how to perform a dietary assessment and basic nutrition counseling. Dietary assessment was conducted according to the USDA’s Automated Multiple-Pass Method (AMPM) [37,38]. The session also outlined the 5 *A*s model—a patient-centered framework for discussing behavioral changes—that was originally developed for smoking cessation but has been more recently adapted for use with patients with obesity [39].

#### 2.1.3. A Culinary Medicine Crash Course

The final session employed culinary medicine education in a virtual teaching kitchen, described as “the previously missing laboratory portion of the historically didactic nutrition curriculum” [22]. This session aimed to serve as a translational, kinesthetic learning activity in this way. Students were asked to cook together synchronously in Zoom breakout rooms.

Study author N.W. (a trained chef) first developed a plant-based recipe by modifying one from a previously published culinary medicine curriculum [40]. This recipe for “Smoky Beans and Rice” (Supplementary S3) features ingredients that are easily accessible and familiar to a variety of cultural and regional backgrounds. The cost per serving was calculated to be $1.72. As this curriculum was taught in a virtual teaching kitchen, participants were provided a gift card for $12.50 and asked to purchase their own ingredients before the session. Basic knife skills and safety techniques were selected as the culinary skills of focus. They are fundamental to preparing health-promoting, plant-based meals and can result in injury if performed incorrectly [41].

The module also aimed to teach students how to read and interpret nutrition fact labels and ingredient lists, define and identify whole grains, minimize dietary sodium while maintaining flavor, and apply the concepts of calorie- and nutrient-density to selecting health-promoting foods. An educational cooking video was scripted and recorded by N.W. before the learning session. The video included N.W. cooking the smoky beans and rice recipe while giving step-by-step instructions and demonstrations to guide participants. Intermittent cues to pause the video were included to allow participants time to complete each recipe step before proceeding with the video. Including graphics- and text-based visual learning tools, the video was edited by a professional video editor whose work was supported by institutional grant funds obtained by the study team.

### 2.2. Curricular Implementation

**Figure 1:**
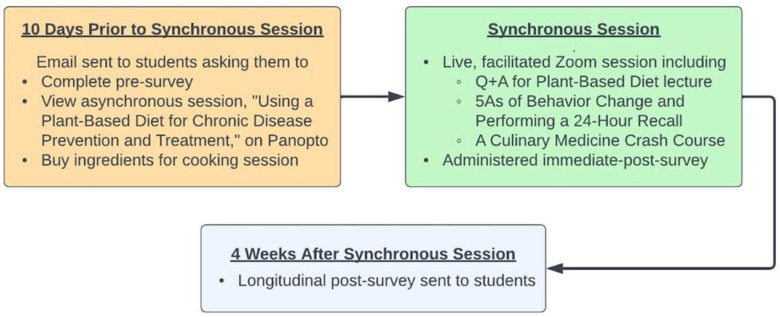
Flow Chart of Curriculum and Survey Implementation.

The session was formatted to fit the pre-existing curricular structure of the YPAOP curriculum, a hybrid of virtual classes and in-person rotations. Participation in the curriculum was required, though study participation was optional. All educational activities in our curriculum occurred remotely. Figure 1 outlines the study timeline.

Ten days before the synchronous session, students received an email with an overview of the curriculum and links to the pre-survey and first module. The first module, “Using a Plant-Based Diet for Chronic Disease Prevention and Treatment”, was delivered asynchronously using Panopto, a video streaming service that incorporates embedded quizzes. Participants were asked to complete the pre-survey before viewing the lecture and were given access to the lecture regardless of study participation.

The synchronous sessions were delivered live using Zoom video conferencing software (version 5.9.6). Two identical three-hour sessions (Supplementary S2) were offered on two days, with half the class assigned to each. This breakdown resulted in a maximum of 40 people per session to promote interaction and engagement. The session began with 30 min of reflection and solicitation of questions about the content covered in the asynchronous WFPB diet lecture. This lecture was followed by the second and third modules (one hour each).

For the first half of the dietary assessment and counseling module, a PowerPoint presentation was used to outline the USDA’s AMPM and the 5 *A*s of behavior change. Then, students were shown a pre-recorded video of a standardized patient case developed by study author J.M. that outlined a clinician (study author B.B.) performing the AMPM and the 5 *A*s with a patient (played by J.M.) to help learners identify the steps to apply this strategy with their patients. After viewing the video, students were divided into triads in Zoom breakout rooms for a case-based exercise. Students took turns role-playing as the patient, clinician, and observer, using case information they received before the session. Session facilitators (J.C., N.W., S.N., and K.G.) were present in breakout rooms to offer feedback. After the role-play activity, there was a group debrief and reflection with time for questions.

For the culinary medicine session, participants were assigned to Zoom breakout rooms. One study team member was the facilitator for each breakout room, showing the video, answering questions, providing supplementary information, and encouraging discussion. Students watched the instructional video over Zoom together and cooked the recipe from their own home kitchen while sharing their progress through their cameras. After participants finished the video and their cooking, the study team members closed the breakout rooms, and everyone gathered back into one virtual teaching kitchen. There was a short debrief led by N.W. as students ate their meals together virtually. Students who participate in culinary medicine sessions in in-person teaching kitchens have been shown to relish this opportunity to ‘break bread’ with each other [42], so the study team wanted to recreate this experience for the students as part of their virtual teaching kitchen experience.

The final 30 min included a session wrap-up and time allotted for the immediate post-survey. Students were asked to reflect on the curriculum and share one learning point. They were also given time to ask final questions. After both sessions and regardless of their participation in the study, all trainees received access to the entire curriculum and references.

### 2.3. Study Participants

**Figure 2:**
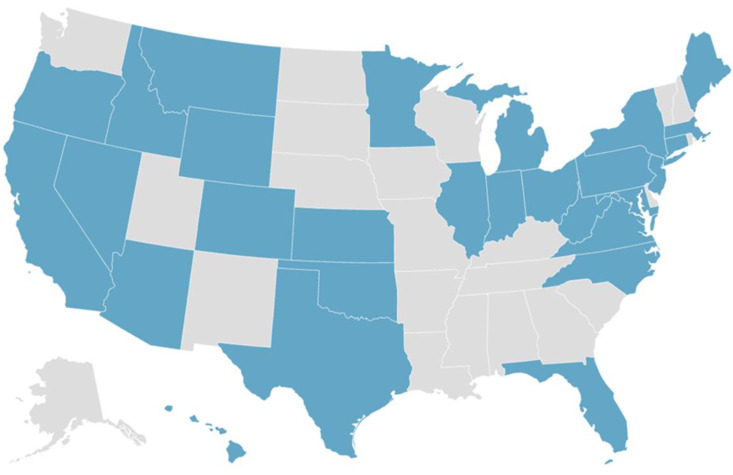
Geographic Distribution of Yale PA Online Program students.

YPAOP students pursue training from their home communities and are located throughout the United States. The 80 YPAOP students in the study cohort represent 28 unique states, as shown in the map in Figure 2.

Table 1 outlines the class profile of the 80 total YPAOP students who received the curriculum. The program collects this data—independently of this project—at the time of matriculation and represents all students in the included cohort, not only those who participated in the study. Of the 80 participants in this study, 34 were first-generation college students. Twenty-eight resided in health-professional-shortage areas, 28 in cities, 34 in suburbs, 5 in towns, and 13 in rural communities. Students came from various clinical backgrounds, ranging from combat medics and paramedics to physical and respiratory therapists.

The majority of the YPAOP didactic curriculum (52 weeks) is completed in an asynchronous, virtual lecture format with the addition of small, synchronous group problem-based learning (PBL) sessions during approximately half of these weeks. Additionally, students attend two one-week-long immersions on Yale’s campus during their didactic year, practicing history-taking and physical exam skills, cadaver dissection, and clinical skills. After completing the didactic year, students complete 16 months of clinical rotations in clinics and hospitals located near their home communities.

This nutrition curriculum was implemented during the third month of didactic training (March 2022). A total of 80 first-year YPAOP students were eligible for participation at the time of the study. All were required to participate in this curriculum as part of their program requirements, but participation in the study component was voluntary. Because the curriculum itself was mandatory, there was no control group, and instead, we compared each participant to themselves across time points. There were no exclusion criteria for either participation or survey completion.

### 2.4. Measurement

Surveys were created using Qualtrics Online Survey Software (https://www.qualtrics.com/, accessed on 20 April 2022). The pre-survey contained questions assessing student demographic and background data, prior training in nutrition, attitudes toward nutrition, and personal dietary behaviors [43]. Students were asked about prior formal nutrition training and self-directed hours spent learning nutrition, which may lead to different attitudes and knowledge on the subject matter.

Items assessing attitudes and confidence used a five-point Likert scale (1 = strongly disagree, 5 = strongly agree). These items assessed confidence in dietary assessment and counseling, food-label reading, and preparing healthy meals. Additional questions assessed attitudes toward the importance of nutrition in chronic disease management, patients’ ability to change their diets, the role of primary care providers in providing dietary advice, and whether diabetes and coronary artery disease can be reversed with dietary changes alone.

Questions were included about students’ personal dietary behaviors, as prior literature has shown the relationship between these behaviors and counseling practices [30,31,32] and that culinary and nutrition education may be able to modify learners’ dietary behaviors [44]. Students were asked to rate their personal nutrition on a 1–10 scale (10 being the healthiest) and select the weekly frequency with which they ate from 12 different food groups (non-starchy vegetables, legumes, dairy products, fish and seafood, etc.) and how often they used different food preparation methods (home-cooked, pre-prepared, or restaurant meals) given five categories ranging from “0” to “7 or more” times per week.

All surveys contained multiple-choice questions to assess students’ knowledge of clinically relevant plant-based nutrition, both generally and relating to addressing cardiovascular disease and type 2 diabetes mellitus. Twenty clinical vignette-style knowledge questions were developed based on session learning objectives (Supplementary S1) using best practices from the National Board of Medical Examiners (NBME) [45]. Through an iterative process with input from subject matter experts in nutrition, lifestyle medicine, and medical education, these questions were consolidated into ten questions included in the final survey (Supplementary S4). Knowledge scores were calculated by determining the number of correct questions out of the total and converting this to a percentage.

The post-survey reassessed students’ attitudes toward nutrition and personal dietary behaviors immediately (“immediate-post”) and four weeks (“delayed post”) after the curriculum was administered. It also included questions regarding satisfaction with each aspect of the curriculum utilizing a five-point Likert scale and free-text responses.

#### Summary of Outcome Measures

1. Change in clinically relevant plant-based nutrition knowledge (percentage of correctly answered knowledge questions);
2. Self-reported attitudes about the role of nutrition in addressing chronic disease in the ambulatory setting (five-point Likert scale);
3. Self-reported confidence in dietary history-taking and counseling (five-point Likert scale);
4. Self-reported personal dietary rating (0–10) and dietary habits (frequency of consumption of each food group via five categories);
5. Satisfaction with each aspect of curriculum (five-point Likert scale and free text response).

Data were analyzed in aggregate, and individual questionnaire scores were not disseminated. Aside from unique identifiers that allowed for the pairing of responses across the three surveys, no individual identifying information was collected in this study.

### 2.5. Data Analysis

Except for Table 2, only participants who finished all three surveys were included in the data analysis. Baseline demographics and prior nutrition knowledge were summarized by count and percentage. Responses to questions about confidence, attitudes, and personal dietary behaviors were dichotomized to “Agree” (a composite of “somewhat agree” and “strongly agree”) and “Other” (a composite of “strongly disagree”, “somewhat disagree”, and “neither agree nor disagree”) to compare them at different time points. Then, McNemar’s test was applied to study if and how participants’ answers shifted after the intervention. A linear mixed effects model was implemented to estimate the marginal means of total knowledge scores at each time point and the change between time points after adjusting for personal nutrition rating in the pre-survey. A power analysis was not performed a priori due to a pre-defined pool of 80 students and an inability to increase sample size beyond planned reminders for students to complete surveys. All analyses were conducted in R [46].

## 3. Results

### 3.1. Participants

All 80 didactic-year YPAOP students attended the synchronous sessions. Of these, 73 (91%) completed the pre-survey, 76 (95%) completed the immediate-post-survey, 42 (52.5%) completed the delayed post-survey, and 38 (47.5%) completed all three surveys. Table 2 shows the baseline demographic characteristics of those who completed all three surveys compared to those who completed the pre-survey only. Those who did not complete all three surveys were generally younger and had a more even age distribution than completers.

Satisfaction with each lecture, defined as a response of either “somewhat satisfied” or “extremely satisfied”, was high across all three curricular sessions. For the 75 students who completed the post-survey, satisfaction rates were 89% for the plant-based diet lecture, 95% for the lecture on nutrition counseling in primary care, and 92% for the culinary medicine workshop. Ninety-three percent, ninety-nine percent, and ninety-nine percent of students believed that each lecture, respectively, should remain part of the YPAOP curriculum with “slight” or “no” changes.

### 3.2. Knowledge

**Figure 3:**
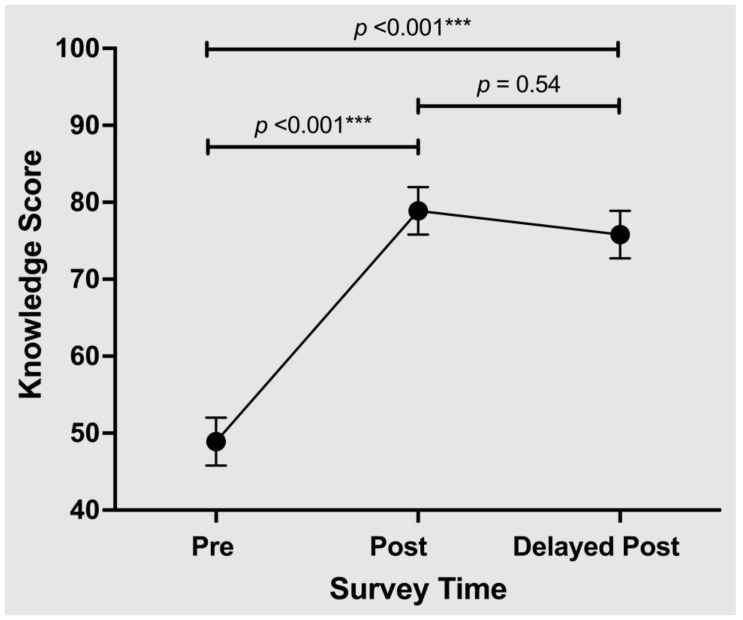
Changes in Nutrition Knowledge Score. *** *p* ≤ 0.001.

Knowledge scores at the three survey time points are shown in Figure 3.

The average pre-test knowledge score was 48.9% (95% CI: 42.8–55.1%). Compared to this baseline, knowledge scores increased by 30 percentage points (95% CI: 22.9–37.1 percentage points) to 78.9% immediately post-curriculum (*p* < 0.001) and by 26.8 percentage points (95% CI: 19.7–33.9 percentage points) four weeks after the curriculum (*p* < 0.001). There was a non-significant decrease in scores between the immediate post-test and delayed post-test (*p* = 0.54).

### 3.3. Attitude, Confidence, and Personal Dietary Behaviors

Table 3 shows the proportion of respondents who, based on dichotomization, agreed or disagreed with attitudinal, confidence, and personal dietary behavior-related questions changes before (“pre”), immediately after (“post”), and four weeks after (“delayed post”) the curriculum.

**Figure 4:**
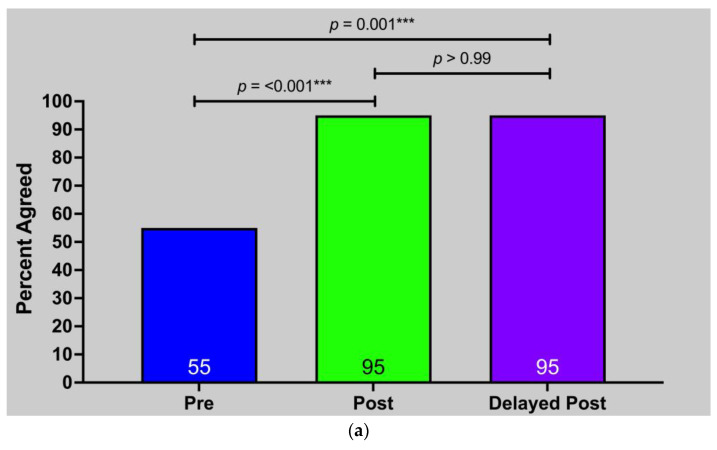

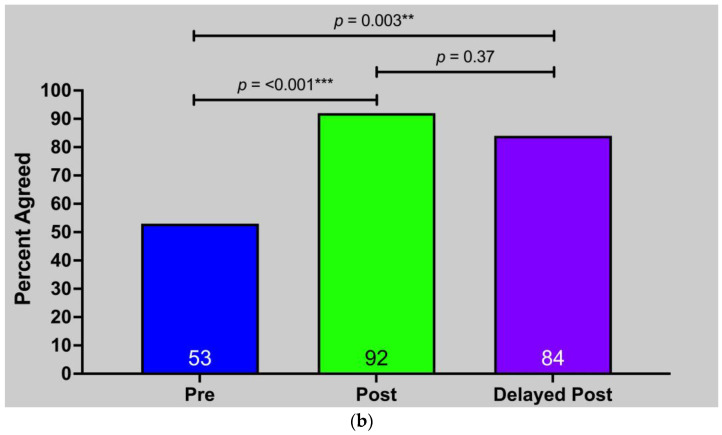
PA Student Confidence in Nutrition Skills Before and After Curriculum: (**a**) Confidence in dietary history taking; (**b**) Confidence in knowledge to counsel patients about nutrition. ** *p* ≤ 0.01 *** *p* ≤ 0.001.

YPAOP students had significantly greater confidence in dietary history-taking both immediately (55% vs. 95%, *p* < 0.001) and four weeks after the curriculum (55% vs. 95%, *p* = 0.001) (Figure 4a). Similar trends were observed for confidence in nutrition counseling (53% vs. 84%; *p* = 0.003) (Figure 4b). These measures had no significant differences between the post and delayed post time points.

**Figure 5:**
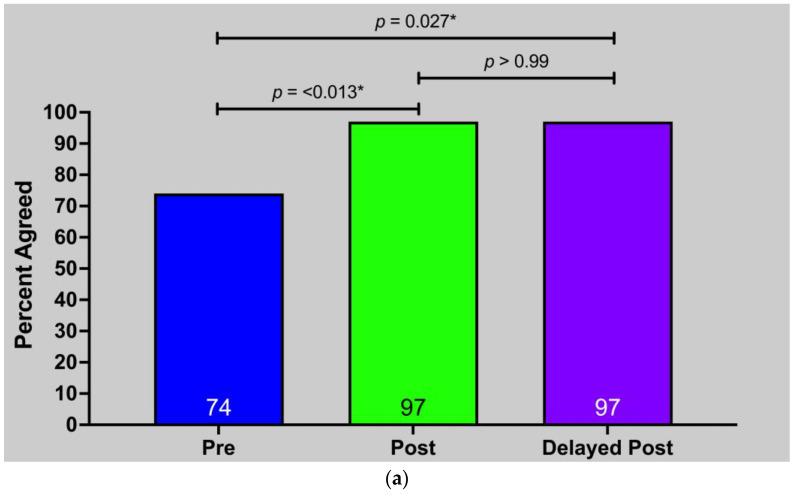

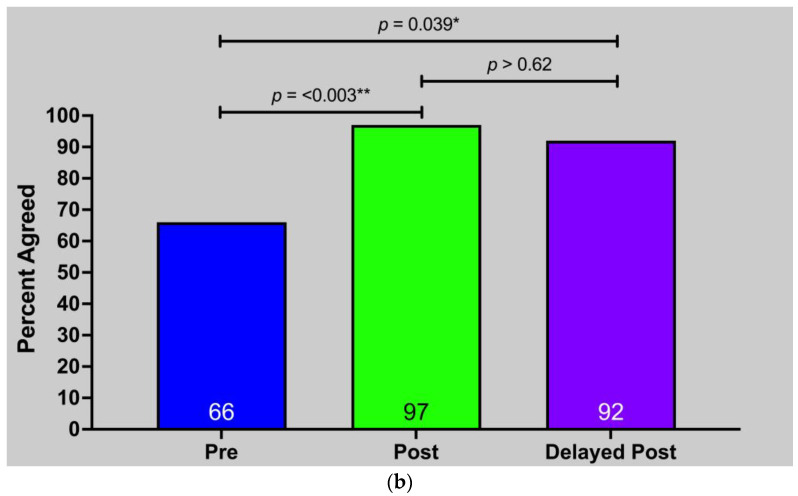
Attitudes toward dietary reversal of chronic disease: (**a**) Belief in inducing diabetes remission with dietary modifications only; (**b**) Belief in inducing coronary artery disease reversal with dietary modifications only.* *p* ≤ 0.05, ** *p* ≤ 0.01.

After participating in the curriculum, students were more likely to agree dietary interventions alone can lead to chronic disease reversal (Figure 5).

There were no changes in students’ attitudes towards the importance of nutrition in chronic disease care or curricular time, personal sense of responsibility to perform nutrition counseling, perceptions of patients’ ability to change dietary behaviors, or the role of primary care providers in giving dietary advice. There were also no significant changes in confidence in and attitudes towards personal healthy dietary habits, types of food consumed, or food-preparation methods.

## 4. Discussion

Our virtual nutrition curriculum was associated with positive increases in clinically relevant plant-based nutrition knowledge, attitudes toward nutrition, and confidence in dietary history-taking and counseling skills among PA students. These benefits persisted four weeks after learners experienced the curriculum. Our study represents an important step forward in nutrition education. It incorporated culinary medicine in a virtual teaching kitchen as part of a virtual PA program, representing a diverse student body. To the authors’ knowledge, this is the first nutrition curriculum using hands-on cooking instruction that has been evaluated in an online PA program. We found that a virtual culinary experience positively impacted trainee attitudes and knowledge.

A previous multi-site trial demonstrated the superior effectiveness of a hands-on culinary medicine course compared to traditional nutrition education for improving attitudes towards and competency of trainees in lifestyle-related counseling [47]. Recent studies have shown similar effects of culinary medicine training in virtual teaching kitchens [48]. While these studies featured multi-session curricula, our findings suggest that even one-time interventions can have positive impacts on trainee outcomes, in line with the findings of a previous study [49]. There were no significant changes in participants’ dietary habits in this study, contrasting with previous studies showing improvements in dietary behaviors [25,47,50]. However, these studies involved more intensive and longitudinal curricula, which are likely to affect personal dietary behaviors more significantly than our four-hour, one-time intervention.

We used hands-on cooking instruction to solidify and extend participants’ nutrition knowledge. The interactivity of our session also served to engage learners. Culinary medicine, as a recent innovation in medical education, has previously been available only in communities and institutions with a physical teaching kitchen. Although teaching kitchens are becoming more prevalent, largely through the advocacy and collaboration of organizations such as the Teaching Kitchen Collaborative [51], they are still quite uncommon. During the COVID-19 pandemic, physical teaching kitchens could not host in-person learning. As a result, programs such as ours resorted to using virtual options, and virtual teaching kitchens proliferated.

Recent research has demonstrated that virtual teaching kitchens, like the one used in this study, are effective and engaging, similar to their in-person counterparts [48,52,53]. In addition to cooking in their own kitchens using their own equipment, learners are also tasked with acquiring their own ingredients. These acts of obtaining food in their local communities and preparing the food in their home kitchens serve to increase self-efficacy, perhaps even beyond the capability of in-person teaching kitchens. Dedicated in-person teaching kitchens are resource-intensive, have space limitations, and often require philanthropic support to get established and maintain programming [27]. Virtual teaching kitchens, however, only require internet access, have no limitations on the number of participants, and can reach patients and learners in rural and underserved communities with limited resources, such as those of many of the learners in this study. These characteristics allow virtual teaching kitchens to increase scalability and access to culinary medicine interventions. The apartment kitchen of N.W. served as the virtual teaching kitchen in this study, with the addition of only an iPhone and tripod stand—a relatively low overhead for an intervention that impacted learners across the U.S.

This curriculum’s culinary medicine session was unique in that it employed a hybrid learning structure. Most virtual teaching kitchens involve an instructor cooking a recipe and teaching live as learners watch and cook along. This study featured a pre-recorded video to provide standardized learning content, while synchronous, hands-on cooking with live facilitator feedback replicated the communal, interactive benefits of physical teaching kitchens. Culinary medicine sessions taught in this format need not have a chef or other culinary professional present to teach the content; only a facilitator is required. Using durable multimedia educational materials such as these allows for the scalability of culinary medicine and nutrition education in virtual teaching kitchens in communities and institutions lacking the trained staff needed to teach these sessions.

Our curriculum focused on tangible, clinically relevant assessment and intervention skills (i.e., dietary history-taking, counseling, and cooking). It used breakout rooms to practice skills with guided observation and real-time expert feedback. We found positive impacts on nutrition knowledge due to this approach. This approach contrasts with much of the nutrition education in medical training, which predominantly focuses on biochemistry and rare nutritional deficiencies rather than the clinical application of nutrition [54] and is frequently taught asynchronously via online lecture modules [55]. Additionally, this curriculum helps address recent calls to action for the medical profession to promote plant-based nutrition [56].

Our study had several limitations. Only 38 of the 80 students (47.5%) responded to all three surveys and were included in data analysis. This was largely due to a poor response rate for the delayed post-survey (52.5%), which students needed to complete on their own, compared to pre- (91%) and post-surveys (95%), which were administered along with the curriculum. Response rates for health-profession-education survey-based research can vary widely, ranging from 26 to 100% in a prior study [57]. The relatively low response rate in our study subjects our findings to non-response bias. Additionally, the baseline demographic data of those who responded to all three surveys vs. those who did not showed differences in the proportion of students who held a prior nutrition degree and distributions of age, self-study, and structured nutrition hours. This indicates that respondents may have been more motivated to engage in nutrition education, which may have overestimated positive attitudes toward nutrition and the ability to learn and retain information. Future studies can address non-response bias using mandatory rather than voluntary participation [58] or collecting additional data from non-respondents to conduct non-response bias analyses [59,60].

The study may have also been subject to social desirability bias, where students dishonestly answered questions about attitudes and behaviors to adhere to social norms [61]. One of the study authors (S.N.) was the director of didactic education at the time of the study, which may have motivated students to report more favorable attitudes and interests than they otherwise would have. However, it was made clear that while participation in the curriculum was mandatory, participation in the study was optional and would not affect grades. Future studies can minimize the effects of social desirability bias by using validated scales to test and control for social desirability and collecting data beyond self-report alone [62]. While survey questions assessed changes in nutrition knowledge and confidence in related counseling skills, objective behavior change metrics were not collected from students or their patients. This was because students do not regularly see patients during their didactic year. Future studies that assess students during different phases of clinical training would allow for the use of both subjective and objective assessments.

Finally, rather than having a control or comparator group in this study, we compared each participant to themselves at each time point. This was undertaken because the curriculum was required for all didactic-year YPAOP students. However, the lack of formal control limits internal validity and prevents drawing conclusions about the success of this curriculum compared to other educational modalities. Future interventions should use a control group or more rigorous quasi-experimental methods, such as a single-case experimental design [63].

There are also several directions for further research. While multiple surveys were used in this study, longitudinal surveys to follow these students into their clinical practice and measure the potential lasting impact of this curriculum could provide valuable insight. Future studies could also use tools such as objective structured clinical exams (OSCEs) to better assess the application of clinical skills compared to the self-reported assessments used in this study [64]. Future research should also investigate potential associations between curricular outcomes and factors such as prior total patient care hours, prior professional degree, or residing in a health provider shortage area (HPSA), which would help fill an important gap in the existing literature.

## 5. Conclusions

Students in an online physician assistant school had increased nutrition knowledge, confidence in nutrition skills, and perceived importance of clinical nutrition after receiving virtual nutrition education and a culinary medicine curriculum. The intervention was well-received by learners and the administration. The curriculum, owing to its novel hybrid asynchronous–synchronous structure and use of a virtual teaching kitchen, offers exciting opportunities for expansion and scalability.

## Figures and Tables

**Table 1:** Class Profile for Yale PA Online Program Matriculating in 2022.

Characteristic	Mean/n (%) ^1^
Age (years)	31.4
Gender	
Female	58 (72.5%)
Male	15 (18.8%)
No Response	7 (8.8%)
Race ^2^	
White	54 (67.5%)
Asian	11 (13.6%)
Black	9 (11.3%)
American Indian	3 (3.8%)
Other (non-specified)	1 (1.3%)
No Response	7 (8.8%)
First Generation College Student	
Yes	34 (42.5%)
No	46 (57.5%)
Health Provider Shortage Area	
Yes	28 (35.0%)
No	52 (65.0%)
Region	
City	28 (35.0%)
Suburban	34 (42.5%)
Town	5 (6.3%)
Rural	13 (16.3%)
Military Affiliation ^3^	
Yes	9 (11.3%)
No	60 (70.5%)
No Response	11 (13.8%)

^1^ n/N (%), N = 80. ^2^ Participants were allowed to select more than one option so percentages can total > 100%. ^3^ A veteran, member of the reserves or National Guard, or a military dependent.

**Table 2:** Baseline demographic characteristics.

Characteristic	%		
	All ^1^ (N = 73) ^1^	Completers ^2^ (N = 38)	Non-Completers ^3^ (N = 35)
Age (yrs)			
21–25	15%	5%	26%
26–30	33%	37%	29%
31–35	30%	34%	26%
>35	22%	24%	20%
***Prior Nutrition Education***			
Nutrition Degree			
Yes	37%	47%	26%
No	63%	53%	74%
Number of Structured Nutrition Hours			
1–5	44%	53%	34%
6–10	15%	11%	20%
11–15	8.2%	7.9%	8.6%
16–20	8.2%	7.9%	8.6%
>21	25%	21%	29%
Number of Self-Study Nutrition Hours			
1–5	22%	32%	11%
6–10	32%	18%	46%
11–15	14%	16%	11%
16–20	4.1%	5.3%	2.9%
21+	29%	29%	29%

^1^ Participants who completed pre-survey only. ^2^ Participants who completed all three of pre-, post-, and delayed-post surveys. ^3^ Participants who did not complete all three of pre-, post-, and delayed-post surveys.

**Table 3:** Change in PA Online Program Student Confidence, Attitude, and Personal Dietary Behaviors.

Characteristic ^1^	n (%) ^2^			*p*-Values ^3^	
	Pre (Time 1)	Post (Time 2)	Delayed-Post ^4^ (Time 3)	Time 1 → 2	Time 1 → 3
***Confidence***					
Dietary History Taking				<0.001 ***	0.001 ***
Agree	21 (55%)	36 (95%)	36 (95%)		
Disagree	17 (45%)	2 (5.3%)	2 (5.3%)		
Knowledge to Counsel Patients				<0.001 ***	0.003 **
Agree	20 (53%)	35 (92%)	32 (84%)		
Disagree	18 (47%)	3 (7.9%)	6 (16%)		
Ability to Cook Healthy Meal				0.48	>0.99
Agree	35 (92%)	37 (97%)	35 (92%)		
Disagree	3 (7.9%)	1 (2.6%)	3 (7.9%)		
Read Nutrition Labels				0.13	0.68
Agree	33 (87%)	37 (97%)	35 (92%)		
Disagree	5 (13%)	1 (2.6%)	3 (7.9%)		
***Attitudes***					
Importance of nutrition for preventing and treating chronic disease				>0.99	>0.99
Agree	36 (95%)	38 (100%)	37 (97%)		
Disagree	2 (5.3%)	0 (0%)	1 (2.6%)		
Learning nutrition is effective way to spend curricular time				0.48	>0.99
Agree	35 (92%)	38 (100%)	37 (97%)		
Disagree	3 (7.9%)	0 (0%)	1 (2.6%)		
Professional responsibility to counsel patients on nutrition				>0.99	>0.99
Agree	36 (95%)	38 (100%)	37 (97%)		
Disagree	2 (5.3%)	0 (0%)	1 (2.6%)		
Nutrition counseling is role of dietitians not primary care providers				0.21	0.043 *
Agree	13 (34%)	18 (47%)	20 (53%)		
Disagree	25 (66%)	20 (53%)	18 (47%)		
Patients expect primary care provider to be healthy eating role model				0.62	0.62
Agree	33 (87%)	36 (95%)	36 (95%)		
Disagree	5 (13%)	2 (5.3%)	2 (5.3%)		
Dietary counseling can motivate patients to change diet				0.37	0.68
Agree	33 (87%)	37 (97%)	36 (95%)		
Disagree	5 (13%)	1 (2.6%)	2 (5.3%)		
Dietary changes alone can induce diabetes remission				0.013 *	0.027 *
Agree	28 (74%)	37 (97%)	37 (97%)		
Disagree	10 (26%)	1 (2.6%)	1 (2.6%)		
Dietary changes alone can induce coronary artery disease reversal				0.003 **	0.039 *
Agree	25 (66%)	37 (97%)	35 (92%)		
Disagree	13 (34%)	1 (2.6%)	3 (7.9%)		
***Personal Dietary Habits***					
Personal Healthy Diet Importance				0.48	>0.99
Agree	36 (95%)	38 (100%)	37 (97%)		
Disagree	2 (5.3%)	0 (0%)	1 (2.6%)		
Interest in Learning More About Nutrition				0.37	0.68
Agree	34 (89%)	37 (97%)	36 (95%)		
Disagree	4 (11%)	1 (2.6%)	2 (5.3%)		
Interest in Dietary Improvement				0.023 *	0.29
Agree	30 (79%)	37 (97%)	34 (89%)		
Disagree	8 (21%)	1 (2.6%)	4 (11%)		
Personal Nutrition Rating (1–10, 10 = high)				>0.99	0.50
≥5	29 (76%)	30 (79%)	32 (84%)		
<5	9 (24%)	8 (21%)	6 (16%)		

^1^ Questions were asked using a 5-point Likert Scale (1 = strongly disagree, 5 = strongly agree) unless otherwise specified. Answers were dichotomized to agree (4–5) or disagree (1–3). ^2^ n/N (%), N = 38. ^3^ McNemar’s chi-squared test with continuity correction. ^4^ Delayed-post refers to delayed post-survey 4 weeks after the synchronous session. * *p* ≤ 0.05 ** *p* ≤ 0.01 *** *p* ≤ 0.001.

## Data Availability

The datasets used in this study are available from the corresponding author upon reasonable request.

## Supplementary Materials

The following supporting information can be downloaded at: https://www.mdpi.com/article/10.3390/nu15194166/s1. Supplementary S1: Curriculum Learning Objectives. Supplementary S2: Curriculum Schedule Sent to Students. Supplementary S3: Smoky Beans and Rice Recipe. Supplementary S4: Knowledge Questions.
