# Supplementary material for: “Zoom”ing to the Kitchen: A Novel Approach to Virtual Nutrition Education for Medical Trainees
Source: Nutrients. 2023 Sep 27;15(19):4166. doi: 10.3390/nu15194166 (PMC10574391; doi:10.3390/nu15194166)
Supplement: Supplementary file 1 [file nutrients-15-04166-s001.zip › nutrients-2589069-supplementary.pdf]

Supplementary Materials

S1: Curriculum Learning Objectives

1. Knowledge
    1.1. Describe the elements and major health benefits of a whole food, plant-based diet (WFPB)
    1.2. Determine the healthful components and patient outcomes associated with DASH and Mediterranean diets
    1.3. Discuss the adverse health effects of dietary animal products
    1.4. Summarize how diabetes and cardiovascular disease can not only be prevented, but treated and even reversed with dietary interventions
2. Attitudes
    2.1. Express appreciation for nutrition as one of the most important modifiable factors for preventing chronic disease
    2.2. Recognize that nutrition is a powerful intervention for treatment and reversal of chronic disease
    2.3. Appreciate that dietary change is something patients strive for, afford, and find the time and skills to do
    2.4. Examine the pivotal role the primary care clinician plays in nutrition counseling
3. Skills
    3.1. Conduct a brief nutritional history in ambulatory settings
    3.2. Counsel a patient on dietary change that better conforms to a WFPB diet
    3.3. Demonstrate basic culinary skills, including knife skills and preparing a basic WFPB-compatible meal

S2: Curriculum Schedule Sent to Students

The live session will proceed as follows:

2:00 - 2:30 PM: Debrief and Question/Answer Session on Using a Plant-Based Diet for Chronic Disease Prevention and Treatment with Dr. Justin Charles

- We will reflect and discuss your lingering questions from watching the session on plant-based nutrition

2:30 - 3:30: Nutrition Counseling in Primary Care: The 5 *A*s & Performing a 24-Hour Food Recall with Dr. Katie Gielissen

- This session will cover how to use the 5 *A*s in discussing weight loss and diet with patients and expert tips and tricks to get an accurate 24-hour dietary recall
    - We will split you into breakout rooms at the end of the session to practice your skills in dietary recall and counseling

3:30 - 4:30 PM: Culinary Medicine Crash Course with Chef Dr. Nate Wood

- In this one-hour, hands-on workshop, you'll be learning together about the basics of culinary medicine, reinforcing patient-centric

nutrition knowledge, and cooking a complete Whole Food Plant Based meal from the comfort of your own kitchen

- Please see the recipe for Smoky Beans and Rice linked here and attached so you can acquire your ingredients in advance of the session

4:30 - 5:00 PM: Wrap-Up and Post-Survey

S3: Smoky Beans and Rice Recipe

Serves 4

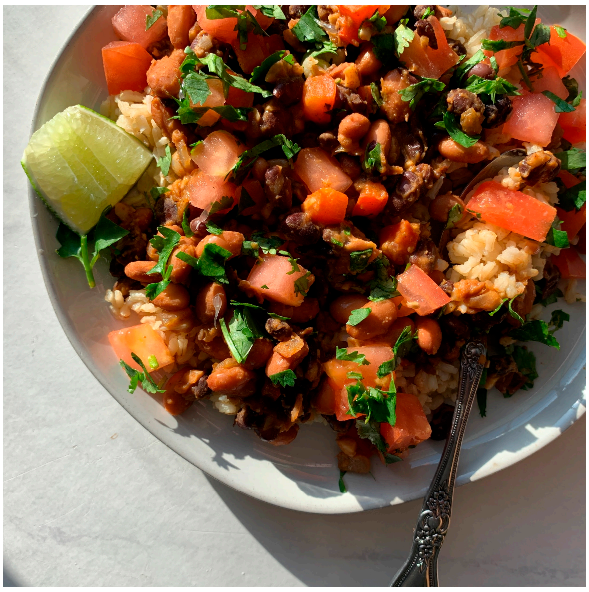

EQUIPMENT:

- Cutting board
- Chef's knife
- Large skillet/sauté pan
- Silicon spatula or wooden spoon

INGREDIENTS:

- Rice:
  - 1 package of microwave-ready brown rice (or other whole grain of your choice)
- Beans:
  - 2 T. low-sodium vegetable broth
  - 1 medium onion, diced
  - 1 medium bell pepper, diced
  - 4 cloves garlic, finely chopped
  - 1 C. low-sodium/no-sodium vegetable stock
  - 1 14.5-oz can black beans, rinsed and drained
  - 1 14.5-oz can pinto beans, rinsed and drained

- 2 t. chili powder
- 2 t. ground cumin
- ½ t. dried oregano
- Black pepper, to taste
- Kosher salt (optional), to taste

- For Serving:
  - ¼ large tomato, diced
  - Juice of ½ lime
  - ¼ C. cilantro, chopped

INSTRUCTIONS:

1. Heat broth in a skillet over medium heat and sauté diced onion and bell pepper, stirring frequently, until just starting to turn soft and translucent, approximately 5 minutes. Add garlic. Continue cooking until the garlic is soft and the onions are just starting to brown on the edges, about 2 minutes more.
2. Add beans, stock, and spices. Season with pepper and salt, if using. Bring mixture to a simmer and cook for 10 minutes, stirring occasionally to prevent sticking. Crush up a few of the beans by smearing them against the side of the pan using the back of your spatula or wooden spoon. Stir to combine.
3. Cook brown rice according to package instructions.
4. If desired, season with more salt and pepper to taste. To serve, spoon ¼ of the bean mixture over ¼ of the rice and top with ¼ C. tomatoes, juice of half a lime, and 1 T. chopped cilantro.

S4: Knowledge Questions
Correct answers are in bold

1. A 53-year-old woman has type 2 diabetes mellitus and an HbA1c of 7.5%. Her diet consists of fruits, vegetables, whole grains, low-fat dairy, and lean poultry or fish. Which of the following dietary changes will most likely reduce her insulin resistance?
   a. Decrease her fruit intake
   b. Decrease her whole grain intake
   c. Decrease her low-fat dairy and lean meat intake
   d. Increase her olive oil intake

2. A 56-year-old man with hypertension, type 2 diabetes mellitus, and coronary artery disease presents to an office visit excited to tell his provider about the dietary changes he has made since his last visit. He bought the following foods: Impossible/Beyond Plant-Based Burger, Vegan Soy Cheese, Vegan Pea Protein Powder, Tofu, and fruit

cocktail. Which of these foods are part of a Whole Food Plant Based Diet?

a. Impossible/Beyond Plant-Based Burger
b. Vegan Soy Cheese
c. White potato
d. Fruit cocktail

3. A 45-year-old man presents to his primary care provider to establish care. He has struggled with weight for most of his life and frequently follows fad diets but has recently become more skeptical. He wants to know what approach will most likely help him reach an optimal body weight in a healthy way. Which one of the following eating patterns will most likely help him achieve this goal?
    a. Near elimination of either fat or carbohydrates
    b. Minimizing foods high in calorie density
    c. Avoiding fruits and starchy vegetables
    d. Eating high quantities of saturated fat

4. A 43-year-old unemployed man with type 2 diabetes and stable angina lives with his four children at home and struggles financially since he lost his job two years ago. His diet consists predominantly of fast food, meat, and cheese as this is "all he can afford". He wants to improve his diet but is concerned that fresh fruits and vegetables and organic products are too expensive for him to purchase. Which of the following is the best dietary advice for this patient?
    a. Frozen fruits and vegetables are unhealthy and should be avoided
    b. Purchase pre-packaged meals to help with portion control
    c. Choose salads or the plant-based burger at fast food restaurants
    d. Buy foods like grains, potatoes, and corn in bulk

5. A 55-year-old woman with an extensive family history of coronary artery disease presents to clinic with dyspnea on exertion. This consistently occurs after walking two blocks that has remained stable for the last 4 years. A recent cardiac stress test showed decreased perfusion of the anterolateral wall of the myocardium suggestive of coronary artery blockage. Her provider recommended statin therapy and workup for coronary angiogram, but she declined. She would like to improve her coronary artery disease with dietary interventions. What level of improvement that can occur with implementation of an intensive dietary intervention?

a. Prevention of coronary artery disease, but no improvement pre-existing coronary artery disease
b. Reduction in frequency and severity of angina, without slowing coronary plaque development
c. Slowing the development of new coronary plaques but has no impact on current plaque burden
d. Increase in coronary artery diameter and improvement in myocardial perfusion

6. Which of the following dietary modifications contributes most to the overall benefits of a Mediterranean dietary pattern?
   a. High consumption of low-fat dairy products
   b. High consumption of fish and other seafood
   c. Low consumption of meat products
   d. Low consumption of whole grains and starchy vegetables

7. A 35-year-old healthy, health-conscious woman presents to your clinic for a preventive care visit. She has a strong family history of heart disease and diabetes and wants to avoid developing chronic disease. She read about an exclusively plant-based diet online and wants to discuss this with you. She says, "doc, I know vegetables are good for you, but I have always thought foods like chicken and dairy are healthy! Should I really give up eating animal products?". Which of the following is the best response to her inquiry?
   a. Avoid meat and dairy that is high in saturated fat, which can lead to heart disease, but it is healthy to eat low-fat meat and dairy products
   b. Avoid cooking meat at high temperatures, which creates harmful compounds, like polyaromatic hydrocarbons, but it is healthy to eat meat that is baked or boiled
   c. Exclusively eating plant-based foods will not give you enough nutrients like protein, calcium, and iron, so make sure you eat plenty of poultry and dairy to stay healthy
   d. Exclusively eating plant-based foods can provide all the nutrients you need to be healthy and reduce your risk of chronic disease

8. A 40-year-old man with obesity and pre-diabetes presents to your clinic to discuss weight loss and diabetes prevention. He said he read about the ketogenic diet and was excited to hear how it promotes

weight loss and helps control diabetes. Which of the following is true about the ketogenic diet?

   a. **A ketogenic diet promotes loss of mostly water weight**
   b. A ketogenic diet reduces insulin resistance
   c. A ketogenic diet slows the development of coronary atherosclerosis
   d. A ketogenic diet decreases the risk of developing diabetes

9. A 64-year-old woman with obesity and type 2 diabetes presents to your clinic for her annual visit. She reports that her daughter has been pestering her to eat more whole grains. So far, she has managed to switch from white bread to multigrain bread and from white rice to brown rice. Due to these swaps, which of the following is true about her new dietary habits?
   a. **Her intake of fiber has increased**
   b. Her intake of micronutrients (vitamins and minerals) has decreased
   c. Her intake of energy in the form of carbohydrates has increased
   d. Her intake of fat has decreased

10. A 68-year-old woman with hypertension who presents to your office for ankle swelling. Her history, exam, and diagnostic work-up are suggestive of heart failure with reduced ejection fraction. In addition to starting guideline-directed medical therapy, you recommend that she adopt a whole food plant-based diet and limit her intake of sodium. How could she modify her cooking to help her reduce her sodium intake?
   a. **Cook with acid-containing foods, such as citrus juices, and tomatoes**
   b. Use fewer spices and herbs
   c. Use pre-packaged spice blends, such as taco seasoning packets
   d. Avoid sodium-free salt substitutes
